# Discontent in the Mental Hospital Service

**Published:** 1913-12-13

**Authors:** 


### DISCONTENT IN THE MENTAL HOSPITAL SERVICE.

### Combination Among Assistant Medical Officers.

NEW ASSOCIATION TO BE FORMED.

A further stage in the movement for combination among assistant medical officers in public mental hospitals was marked by a meeting of North of England officers held at Manchester on Wednesday last week. The meeting was convened by the Association which has already been formed for the Midlands. The following North of England officers were present: Dr. Wilkins, Winwick; Dr. Clayton, Winwick; Dr. Devine, Wakefield; Dr. Walker, Menston; Dr. Kirwan, Menston; Dr. Stephenson, Prestwich; Dr. T. Hibbert, York City Mental Hospital; Dr. Firth, Wadsley; Dr. Mathieson, Wadsley; Dr. Smith, Whittingham; Dr. Denning, Rainhill; Dr. Fehily, Rainhill. The Midland Association was represented by Dr. F. J. Stuart, Berrywood; Dr. Cormac, Macclesfield; Dr. Shaw, Stafford; and Dr. Howard, Chester. Dr. Devine was appointed chairman of the meeting, which was held with a view to the formation of an Association for the North of England.

After a few introductory remarks by the Chairman, Dr. Stuart, of the Midland Association, described the objects which that body was intended to promote. He pointed out that the advancement or promotion of assistant medical officers in mental hospitals had nothing whatever to do, under present conditions, with merit or energy. It was entirely a question of good fortune, depending chiefly upon the promotion of the medical officers' superiors. With regard to pay, Dr. Stuart stated that there were many senior assistant medical officers who, after many years' asylum experience, were receiving less than was being paid by the Lancashire County Asylums for men with no asylum experience at all. Moreover, in many cases, even after ten and fifteen years' service, assistant officers were not allowed to marry. Dr. Stuart instanced the case of a man who, having been permitted to marry, found himself five years afterwards with no accommodation for a wife. In another case a man, having got married, was forced to live in the asylum, whilst his wife had to live outside the institution. This, said Dr. Stuart, was taking place at a time when the housing of the labouring classes was occupying the serious consideration of men of all parties in this country.

The meeting having discussed the matter in private, it was decided to form an Association for the North of England, and representatives were elected to arrange terms of amalgamation with Assistant Medical Officers' Associations in different parts of the country.

Dr. Stuart (Berrywood) was appointed secretary of the new Association.

### MEETING AT BRISTOL.

On Thursday last week a meeting of assistant medical officers of South Wales and the West of England was also held in private at Bristol, which Dr. Stuart addressed on similar lines, and, we understand, terms of amalgamation with the other Associations of Assistant Medical Officers were discussed, and representatives chosen to arrange them.

### MEETING IN LONDON.

On Friday last week some thirty assistant medical officers of public mental hospitals in London and the district met at Westminster Palace Hotel to discuss combination. This, the fourth, meeting which has been held in connection with the new movement, was also addressed by Dr. F. J. Stuart.

In the course of his speech he referred to the scarcity of applicants for assistant medical officers' posts in mental hospitals as indicating the unpopularity of the service. It was felt that there were no assured prospects and no reasonable hope of marrying or getting a superintendentship. Every University professor warned men against the service, and every medical school regarded it as a refuge for failures. The first steps towards any attempt at improvement were taken when the Medico-Psychological Association suggested diplomas which the Universities granted, but assistant medical officers were hindered from obtaining them because they could not obtain study leave. Two years ago it was recommended at a special meeting of the Association that study leave be granted, and powers were given to the Education Committee to discuss ways and means. But the result was little enough. The Status Committee also had worked well, but though its work had been heavy, Parliamentary sanction would be required for many of the proposals, and in the state of party legislation that meant indefinite delay. In the meantime the service might be said to have broken down. The result was the issue of circular letters from Durham, the taking up of the question in the medical press, and the holding of the Birmingham, Manchester, and Bristol meetings.

There was, indeed, no need to labour old standing grievances. He had to mention only the way in which statements even on scientific subjects by assistant medical officers had to be predigested by superintendents before they were considered worthy to appear. He maintained that science could not progress where young scientists were deprived of free expression of their views. Local "rigging" and dead men's shoes were the only avenues to promotion, with the result that men who were considered worthy to be on the short list for a post worth perhaps £1,200 or more a year, if unsuccessful (as all except one man must necessarily be) had to return to work at £300 or even less, and if married to live apart from their wives. Again as to salaries. So difficult were junior posts to fill that you could not now procure a junior assistant for what senior men appointed in the past are still receiving. At Hereford the senior assistant was getting £180, and till last week the senior under the Lancashire Asylums Board £250. But if it was good economy to pay the superintendents well, it was good economy to pay the assistant medical officers well also. The great disparity between the salaries of superintendents and assistants, he maintained, was bad economy. As to the enforced celibacy, mental hospital committees were proudly boasting that they were providing houses for married attendants when assistant medical officers could not get married at all, and the Lunacy Commissioners actually patted these committees on the back, while assistant medical officers were often badly housed. These committees virtually said, "You may get married if you live apart from your wife," and assistant medical officers had only to combine, so that individuals might act fearlessly, to arouse public opinion, which was now ripe, to drive this state of affairs home at the popular elections of the committeemen, which took place every three years. He did not think any candidate for election would like to fight under such a banner.

The rest of the proceedings were in private, but it was resolved to elect representatives to meet representatives from other parts of the country to set up an Association of Assistant Medical Officers.

